# Platform leadership and subordinates’ proactive career behavior: the role of thriving at work and career centrality

**DOI:** 10.3389/fpsyg.2025.1552925

**Published:** 2025-05-21

**Authors:** Zheng Ji, Kaineng He, Jinyan Zhu, Miao He

**Affiliations:** School of Labor and Human Resources, Renmin University of China, Beijing, China

**Keywords:** platform leadership, proactive career behavior, thriving at work, career centrality, self-determination theory

## Abstract

**Introduction:**

Platform leadership is a type of leadership characterized by building shared career platforms to foster the growth of subordinates, leaders and organizations. Despite its rising popularity, the implications of platform leadership, particularly its impact on employee outcomes, remain largely unexplored. Based on self-determination theory, this paper discussed the mechanisms and boundary conditions of platform leadership on subordinates’ proactive career behavior.

**Methods:**

To examine these effects, three-wave matched data were collected from 294 employees across diverse industries. The authors used hierarchical regression and bootstrapping analyses to test the hypotheses.

**Results:**

The results showed that platform leadership promoted subordinates’ proactive career behavior. Thriving at work mediated this relationship. Additionally, higher subordinates’ career centrality strengthened the role of platform leadership in promoting thriving at work, and also enhanced the indirect effect of thriving at work between platform leadership and proactive career behavior.

**Discussion:**

Our study makes three key contributions to the platform leadership and career development literature. First, it is the first to investigate the mechanism and boundary conditions linking platform leadership to proactive career behavior. Second, the results offer actionable insights for leadership training and development programs. Finally, this research highlights the critical role of platform leadership in enhancing employees’ perceptions of career platform value, thereby fostering long-term organizational growth through sustained proactive engagement.

## 1 Introduction

With the rapid advancement of digital technology, we have entered an era characterized by knowledge, information, and innovation (Min and Kim, 2020). The knowledge economy has fundamentally reshaped expectations for career management, compelling employees to proactively acquire evolving skill sets and assume greater ownership of their professional trajectories (Autor, 2015; Haenggli et al., 2021). In this paradigm, career success largely depends on proactive career behavior—a critical determinant of both individual advancement and organizational adaptability (Meyers, 2020).

Proactive career behavior encompasses a range of intentional actions that individuals undertake to realize career goals (Strauss et al., 2012), representing a direct approach to career advancement (Seibert et al., 2001; Van Hoye et al., 2009). These actions include skill development, career planning, seeking career advice, and networking (Strauss et al., 2012). Previous research showed that proactive career behaviors benefit both individuals and organizations (Wang et al., 2024). Specifically, they enhance employees’ task performance and employability over time (Smale et al., 2019) and contribute to organizational resilience and adaptability (Meyers, 2020). Given these benefits, the study of proactive career behavior is of significant importance for academic inquiry and practical application.

Previous research has primarily focused on examining the impact of individual factors, such as regulatory focus, on proactive career behaviors (Peng et al., 2021). However, leadership, as a key contextual factor, plays a significant role in shaping subordinates’ career behaviors (Zhong et al., 2024). Given the numerous benefits associated with proactive career behavior, the role of leadership in actively promoting subordinates’ career initiatives is critical. This highlights the importance of studying leadership as an antecedent to proactive career behaviors. Therefore, our study aims to explore the influence of platform leadership on subordinates’ proactive career behaviors.

Platform leadership (Hao, 2016), an emerging paradigm, offers a novel lens for such inquiry. With the dynamic nature of organizational environments and the rise of knowledge workers, the knowledge economy has introduced new demands for leadership. The defining feature of platform leadership is constructing a shared platform for career development, which facilitates the growth of subordinates, leaders, and the organization as a whole (Hao et al., 2021). This leadership style emphasizes the interaction between leaders, subordinates, and context, creating space for continuous progress and growth. Its aim is to positively impact both the organization and its members, for example, by fostering subordinates’ innovative behaviors (Hao et al., 2021) and supporting the organization’s sustained competitive advantage (Yang et al., 2022).

Although existing research has explored some aspects of the impact of leadership on subordinates’ career outcomes, such as leader humility (Zhong et al., 2024) and servant leadership (Wang et al., 2019), our study offers a distinct perspective. The uniqueness of platform leadership lies in its ability to create a shared business platform that not only elevates the common goals of departments or teams but also makes employees’ work more challenging and meaningful. This leadership approach significantly influences employees’ career development and satisfaction by aligning personal and organizational goals and fostering intrinsic motivation. Therefore, exploring the relationship between platform leadership and proactive career behaviors represents an exciting avenue for both academic inquiry and practical application, with the potential to illuminate how leaders can effectively foster the career development and success of their teams in the modern workplace.

We hypothesize that platform leadership positively predicts thriving at work, particularly among employees with high career centrality. Drawing on Self-Determination Theory (SDT, Deci and Ryan, 2000, 2004), this study examines how situational factors (platform leadership) and individual differences (career centrality) jointly shape proactive career behaviors through autonomous motivation. Specifically, we propose that platform leadership fosters subordinates’ autonomous motivation by creating value-aligned career platforms, thereby energizing proactive career engagement.

Building on this, the study introduces thriving at work as a mediator, defined as a psychological state in which individuals experience both vitality and learning simultaneously at work (Spreitzer et al., 2005). This state reflects autonomous motivation and psychological growth, and it may mediate the effect of platform leadership on subordinate behaviors. Additionally, individual differences may influence the effectiveness of leadership (Wang et al., 2019). Therefore, this study incorporates career centrality as a moderator, which refers to the extent to which individuals regard career-related matters as personally important (Erdogan et al., 2018). Employees with higher career centrality are more likely to internalize platform leadership’s developmental signals, intensifying its effects on thriving and subsequent career behaviors.

**Figure 1:**
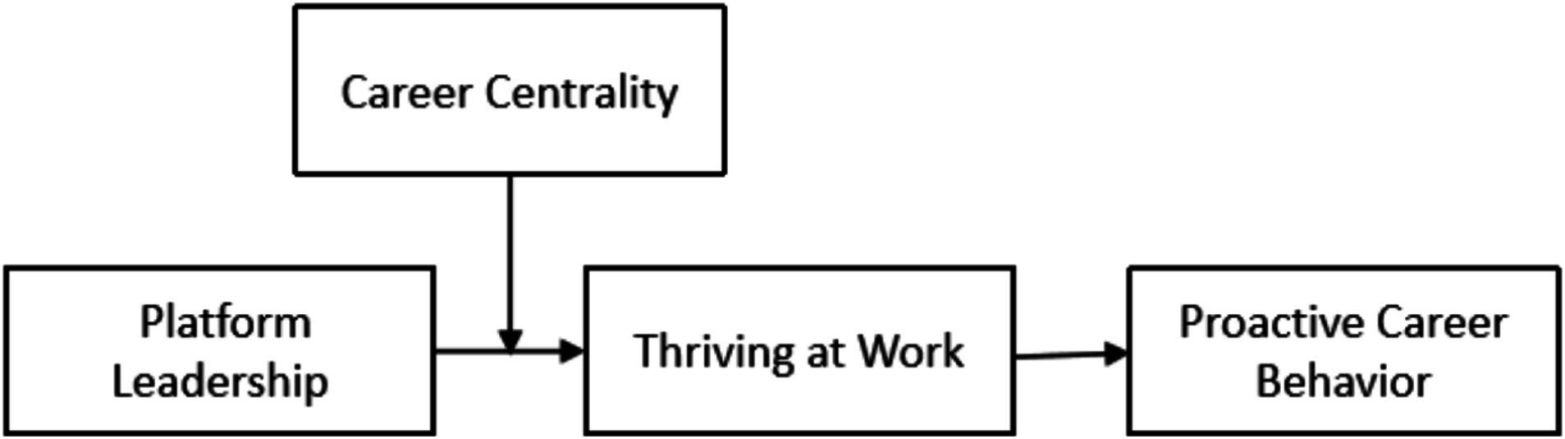
Theoretical model.

In summary, we posit a moderated mediation model. The indirect effect of platform leadership on proactive career behaviors via thriving strengthens as career centrality increases. This research investigates the mechanisms and boundary conditions through which platform leadership influences subordinates’ proactive career behaviors, offering empirical evidence to support the scientific promotion of employees’ career growth and success. The theoretical model is presented in Figure 1.

Our research makes three key contributions. First, this study introduces proactive career behavior as a novel outcome variable to evaluate platform leadership’s effectiveness, thereby expanding the scope of leadership impact research. Distinct from prior focus on innovative behaviors (Hao et al., 2021), proactive career behavior, a person-environment fit mechanism (Grant and Parker, 2009), directly bridges individual career advancement and organizational adaptability. Consequently, this study offers a new theoretical perspective on how leadership styles influence employees’ career development trajectories.

Second, this study pioneers the exploration of platform leadership’s unique role in contemporary career development. While sharing employee development emphases with other leadership paradigms, platform leadership distinctively adapts to dynamic organizational ecosystems through three mechanisms: (a) co-creating career advancement platforms with followers, (b) fostering leader-subordinate-environment synergies via platform expansion, and (c) enabling subordinates to perceive latent growth opportunities within their roles (Hao et al., 2021). This tripartite approach redefines leadership’s capacity to convert environmental complexity into developmental momentum.

Third, drawing on Self-Determination Theory, we unravel the “how” and “for whom” of platform leadership’s effects. By introducing thriving at work as a mediator and career centrality as a moderator, this study shift from asking whether platform leadership matters to explaining its psychological mechanisms and boundary conditions. This dual focus provides new theoretical insights and empirical evidence to clarify the “black box” of platform leadership’s impact.

## 2 Theory and hypotheses

### 2.1 Platform leadership

In the context of the knowledge economy and the internet era, characterized by dynamic organizational environments and the rise of knowledge-based employees, Hao (2016) introduced a novel leadership model known as platform leadership. This model is distinguished by its emphasis on leaders constructing a shared platform for collaborative endeavors, thereby fostering the mutual development of subordinates, leaders, and organizations (Hao et al., 2021).

Platform leadership is a multifaceted construct comprising six dimensions: inclusiveness, personal charisma, change planning, platform construction, platform optimization, and mutual growth (Hao et al., 2021). Inclusiveness refers to a leader’s broad-mindedness when working with others, characterized by their ability to accept diverse opinions and share information, resources, and achievements with team members. Personal charisma describes a leader’s traits such as positivity, optimism, approachability, and kindness, which inspire subordinates to follow platform leadership. Change planning reflects a leader’s capacity to effectively navigate dynamic environments, formulate sound strategies, and ensure the organization achieves its objectives. Platform construction emphasizes a people-centered approach, where leaders create opportunities for subordinates to showcase their talents, support each other, and grow together. Platform optimization focuses on expanding the platform by maintaining and improving it through achievement-oriented initiatives and organizational learning activities. Mutual growth highlights platform leaders’ dedication to the development of both subordinates and themselves, achieved through attention to subordinate growth, self-improvement, empowerment, and the cultivation of interactive relationships that facilitate mutual success and shared progress (Hao et al., 2021).

The uniqueness of platform leadership lies in its ability to build a shared platform for career development, which not only elevates the common goals and collaborative capabilities of departments or teams but also inspires work with greater challenges and deeper meaning, which fundamentally differs from established paradigms like transformational (vision-driven inspiration), servant (altruistic support), or empowering (delegation-focused) leadership. Platform leadership emphasizes mutual fulfillment and growth between leaders and subordinates, focusing on the joint progress of employees, leaders, and the organization. It prioritizes building and optimizing a shared platform, using an inclusive approach to inspire potential in leaders, subordinates, and the organization, which is crucial for resource integration and goal achievement in dynamic environments. While acknowledging the role of “personal charm” and “change planning,” platform leadership centers on platform development and optimization, rather than relying solely on individual leader traits or actions to influence subordinates or drive organizational change.

Platform leadership distinctively fulfills employees’ basic psychological needs through structural mechanisms, contrasting with established styles. For autonomy, it creates bounded agency via career platforms, enabling self-directed growth within strategic frameworks—surpassing empowering leadership’s narrow focus on decision rights. For competence, it implements dynamic resource-matching systems, ensuring sustained skill mastery beyond transactional training in other leaderships. For relatedness, it builds institutionalized reciprocity through collaboration networks, transcending dyadic bonds in transformational approaches. Platform leadership embeds need fulfillment into organizational infrastructure, ensuring resilience during leadership transitions and environmental shifts. This systemic approach offers a novel theoretical lens for sustaining motivation in dynamic workplaces.

### 2.2 Platform leadership and proactive career behavior

As career paths become increasingly ambiguous, individuals are required to take a more active role in ensuring their employability throughout their careers (Fugate et al., 2004). The rise of boundaryless careers has shifted scholarly attention toward how individuals proactively shape their career futures (Seibert et al., 2001). Proactive career behaviors refer to deliberate actions taken by individuals to achieve their career goals (Strauss et al., 2012; Hirschi et al., 2013). Claes and Ruiz-Quintanilla (1998) were the first to integrate early career management behaviors and identified four distinct types of proactive career behaviors: career planning, skill development, seeking advice, and networking behaviors. Regarding leadership factors, prior research has confirmed the positive influence of coaching (Huang and Hsieh, 2015) and managerial support (Noe, 1996) on proactive career behaviors.

This study proposes that platform leadership significantly promotes subordinates’ proactive career behaviors, as detailed below. First, based on self-determination theory, platform leadership satisfies subordinates’ basic psychological needs (Hao et al., 2021). When their growth and self-actualization needs are met, subordinates are motivated and more willing to engage in proactive career behaviors, such as planning their careers and developing their skills. They perceive the meaning of their efforts, as the fulfillment of basic psychological needs stimulates proactive career behaviors.

Second, platform leadership fosters and optimizes career platforms for mutual growth between leaders and employees, effectively enhancing the success of their departments or teams. Under the guidance of strong platform leadership, employees’ career expectations and motivations for self-actualization are heightened. By emphasizing the establishment and optimization of shared career platforms, this leadership style promotes mutual growth and fulfillment between leaders and subordinates. This approach focuses on providing employees with broad development opportunities, stimulating their potential, and satisfying their self-actualization needs. Consequently, employees under this leadership are more likely to actively set and pursue high-level career goals, reflecting increased career expectation values. Influenced by such leadership, subordinates tend to prioritize their career achievements, invest greater effort, and persistently engage in proactive career behaviors. They also become more inclined to seek advice and assistance from colleagues or build interpersonal networks. At the same time, the continuous optimization of career platforms demands higher levels of knowledge and skills from subordinates, necessitating further skill development.

Lastly, platform leadership provides subordinates with sufficient autonomy and a supportive, inclusive work environment to facilitate organizational learning. Leaders who foster a positive learning atmosphere within the organization (Yang et al., 2022) further encourage subordinates to engage in proactive career behaviors.

In summary, platform leadership possesses “people-centered” and “self-actualization” attributes, which effectively stimulate more proactive career behaviors among subordinates. Accordingly, we propose the following hypothesis.

*H1:* platform leadership is positively related with proactive career behaviors.

### 2.3 The mediating role of thriving at work

Thriving at work is a psychological state in which individuals experience both vitality and learning in their work (Spreitzer et al., 2005). Vitality refers to the sense of energy and enthusiasm for work (Nix et al., 1999), while learning represents the feeling of growth and progress achieved through acquiring new knowledge and skills (Edmondson, 1999). These two dimensions, respectively, reflect individuals’ affective and cognitive psychological experiences during personal development. Individuals with a higher sense of thriving at work perceive personal growth and motivation; they are energetic, vibrant, continuously learning, and constantly improving (Spreitzer et al., 2005). They feel passionate about their work, energize themselves and others, and believe that what they are doing will keep improving. This state not only reflects a positive career attitude but also represents a psychological perception of growth—an internal sense of fulfillment and enhanced self-efficacy gained through work experiences.

Individuals with higher levels of thriving at work are not content with the status quo. They are self-directed learners who actively seek opportunities to learn new things and achieve growth. Previous studies have found that leadership styles such as transformational leadership (Hildenbrand et al., 2018), servant leadership (Walumbwa et al., 2018), and authentic leadership (Mortier et al., 2016) influence employees’ thriving at work.

We propose that platform leadership influences subordinates’ thriving at work. First, based on self-determination theory, platform leadership provides subordinates with a stage for career development, fosters mutual growth, and emphasizes the enhancement of subordinates’ capabilities. Through adequate empowerment and resource support, platform leadership enhances subordinates’ sense of control and responsibility at work, thereby fulfilling their need for autonomy. By building a collaborative and supportive career platform, it creates an atmosphere of appreciation and acceptance, satisfying their need for relatedness. Additionally, by offering training and development opportunities, it helps subordinates improve their skills and competencies, fulfilling their need for competence (Hao et al., 2021). By meeting these psychological needs, platform leadership enables employees to internalize external incentives into intrinsic motivation, allowing them to align with organizational goals and transform them into personal career aspirations, thereby enhancing their proactivity and persistence in career development. According to the integrated model of thriving at work and personal growth, the satisfaction of basic psychological needs contributes to an increase in thriving at work (Spreitzer and Porath, 2013).

Second, platform leadership, characterized by broad-mindedness when working with others and traits such as optimism, approachability, and kindness, effectively navigates dynamic environments, inspiring subordinates’ loyalty, energy, and enthusiasm for work, which results in higher vitality. By sharing information and resources, platform leadership establishes a platform where subordinates can showcase their talents, support each other, and grow together. This leadership style continuously maintains and optimizes the platform, focuses on subordinates’ growth, and provides ample empowerment, enabling subordinates to enhance their skills and confidence through the acquisition and application of knowledge, which fosters higher levels of learning.

In summary, platform leadership enhances subordinates’ vitality and learning, thereby increasing their thriving at work. Accordingly, we propose the following hypothesis:

*H2:* platform leadership is positively correlated with thriving at work.

Previous research has demonstrated that thriving at work is positively correlated with various positive behaviors and outcomes, such as job performance, proactive career behavior (Porath et al., 2011), and innovative behavior (Carmeli and Spreitzer, 2009). We propose that employees with higher levels of thriving at work experience a stronger sense of growth and motivation, which drives them to invest more energy in their career development and engage in more proactive career behaviors. Thriving at work reflects an individual’s psychological sense of growth, while proactive career behavior is a self-growth oriented proactive action. Thriving at work can help employees adapt to their work environment and facilitate personal development and growth (Wallace et al., 2016). Employees with higher thriving at work are more likely to actively seek opportunities for learning and growth, foster a positive work atmosphere, and engage in more proactive career behaviors.

Specifically, when individuals experience vitality in their work, they have more energy and motivation to engage in proactive career behaviors. Similarly, when individuals experience learning in their work, they acquire new professional knowledge, which boosts their confidence in undertaking behaviors aimed at self-improvement.

In summary, we argue that platform leadership promotes proactive career behaviors by enhancing subordinates’ thriving at work. Accordingly, we propose the following hypothesis:

*H3:* thriving at work mediates the relationship between platform leadership and proactive career behaviors.

### 2.4 The moderating role of career centrality

Career centrality reflects the extent to which individuals define themselves within a career context (Fugate et al., 2004). Individuals with higher career centrality tend to place greater importance on career-related matters, viewing their careers as sources of purpose and meaning in life (Hirschfeld and Feild, 2000). They are also more likely to make sacrifices in other aspects of life to achieve career advancement (Erdogan et al., 2018). In other words, employees with strong career centrality are more motivated to achieve career success through effort and are more inclined to seek opportunities for career development at work. Such individuals are likely to proactively set career goals, develop career-related skills, and acquire resources for self-improvement to enhance their competence and better meet work demands and career development needs. In contrast, employees with lower career centrality are less likely to exhibit these characteristics or engage in such behaviors and may be less inclined to invest actively in career management activities.

According to self-determination theory, autonomous motivation depends not only on environmental factors but also on internal resources (Deci and Ryan, 2000, 2004). Based on this, we further propose that career centrality, as an individual characteristic, moderates the relationship between platform leadership and thriving at work, which represents autonomous motivation. The effectiveness of platform leadership may depend on the extent to which individuals prioritize their careers as central to their lives. Career centrality reflects how individuals perceive the importance of their careers, aligning well with platform leadership’s emphasis on fostering subordinates’ growth. Specifically, platform leadership provides career-related resources and opportunities by building platforms where subordinates can showcase their talents, support each other, and grow together, as well as by focusing on subordinates’ growth and granting empowerment. Such support is likely to have a stronger positive impact on employees with higher career centrality.

Employees with higher career centrality highly value their professional growth, possess stronger motivation to achieve career success through effort, and seek career development opportunities in the workplace (Wang et al., 2014). These employees benefit more from the support provided by platform leadership, such as a well-structured career platform. When such employees perceive platform leadership, they are more capable of utilizing the career platform support provided by their leaders to actively develop themselves. They view their leaders as pathways and resources for career development, leading to higher levels of thriving at work. Conversely, employees with lower career centrality may weaken the positive impact of platform leadership on thriving at work.

Based on this, we propose the following hypothesis:

*H4:* Career centrality moderates the relationship between platform leadership and proactive career behaviors. Specifically, the relationship is stronger when career centrality is higher.

### 2.5 The moderated mediation model

Based on the reasoning above, we propose an integrated first-stage moderated mediation model (Edwards and Lambert, 2007), suggesting that the mediating role of thriving at work in the relationship between platform leadership and proactive career behaviors is moderated by career centrality. Specifically, the higher the level of career centrality, the more pronounced the mediating effect. To formally test this moderated mediation effect, we propose the following hypothesis:

*H5:* Career centrality moderates the mediating role of thriving at work in the relationship between platform leadership and proactive career behaviors. Specifically, the indirect effect of thriving at work is stronger when career centrality is higher than when it is lower.

## 3 Methods

### 3.1 Sample and procedure

The data for this study was collected from employees of 15 companies located in Beijing, Shandong, Sichuan, and other regions of China. These companies span a range of industries, including real estate, internet technology, finance, and management consulting, providing a representative sample. Data collection occurred in three waves. In the first wave, participants completed questionnaires assessing their direct supervisor’s platform leadership, their career centrality, proactive personality, and demographic information. One month later, a second wave of questionnaires was distributed to measure thriving at work. After another month, a third wave of questionnaires was sent to measure proactive career behaviors.

A total of 400 employees were invited to participate in the first wave of the survey, with 370 completing the questionnaires, yielding a response rate of 92.50%. Among these, 331 valid responses were collected in the second wave, with a response rate of 89.46%. In the third wave, 304 valid responses were received, representing a response rate of 91.84%. After accounting for incomplete data, the final valid sample consisted of 294 responses.

In the final sample, 70.70% were female (208 participants), and 29.30% were male (86 participants). The age distribution was as follows: 9.20% were 25 years old or younger (27 participants), 17.70% were aged 26–30 (52 participants), 27.90% were aged 31–35 (82 participants), 38.80% were aged 36–40 (114 participants), and 6.50% were 41 years old or older (19 participants). Regarding educational attainment, 29.90% held an associate degree or lower (88 participants), 58.50% held a bachelor degree (172 participants), and 11.30% held a master degree or above (34 participants).

The measurement variables in this study were all adapted from validated scales developed in prior international research. The scales were translated into Chinese using the standard translation-back translation procedure (Brislin, 1980).

### 3.2 Variable measurement

Except for platform leadership, all variables in this study were measured using established scales from prior international research. These scales were translated into Chinese using the standard translation-back translation procedure (Brislin, 1980). All scales used a 5-point Likert scale, where 5 indicated “strongly agree” and 1 indicated “strongly disagree.”

Platform Leadership: Measured using the platform leadership scale developed by Hao et al. (2021). A representative item is: “My leader encourages subordinates to continuously seek new ideas and methods when solving problems.” The scale consists of 25 items, with a high level of internal consistency (Cronbach’s *α* = 0.978).

Thriving at Work: Measured using the thriving at work scale developed by Porath et al. (2011). A representative item is: “At work, I feel energized and full of vitality.” The scale includes 10 items, with Cronbach’s α = 0.922.

Proactive Career Behaviors: Measured using the proactive career behaviors scale developed by Strauss et al. (2012). A representative item is: “I develop knowledge and skills that are critical to my future work life.” The scale consists of 13 items, with Cronbach’s α = 0.956.

Career Centrality: Measured using the career centrality scale developed by Erdogan et al. (2018). A representative item is: “My success largely depends on my career achievements.” The scale includes 3 items, with Cronbach’s α = 0.882.

Control Variables: Two types of control variables were included in this study. The first type consisted of employees’ demographic information, including education level, gender, and age, as these factors may influence individuals’ proactive career behaviors. The second type was proactive personality, which refers to an individual’s tendency to take initiative in influencing their surrounding environment (Bateman and Crant, 1993; Crant, 2000). Proactive personality, which significantly impacts proactive career behaviors, was measured using a scale developed by Parker and Sprigg (1999). A representative item is: “If I firmly believe in something, I will do my best to accomplish it, regardless of the likelihood of success.” The scale includes 4 items, with Cronbach’s α = 0.767.

## 4 Results

### 4.1 Confirmatory factor analysis

We conducted a series of CFAs to assess measurement validity. Table 1 summarizes the results. We examined a model containing four factors for the concept of platform leadership, career centrality, proactive career behavior, thriving at work (Model 1). Due to the large number of measurement items and the relatively small sample size, parameter estimation is prone to bias. Following the suggestion of Mathieu and Farr (1991), platform leadership and proactive career behaviors were packaged by dimensions. This correlated four-factor demonstrated good fit: *χ*^2^ = 559, df = 224, *χ*^2^/df = 2.496; comparative fit index (CFI) = 0.942; Tucker–Lewis index (TLI) = 0.934; root mean square error of approximation (RMSEA) = 0.071, standardized root mean square residual (SRMR) = 0.041.

**Table 1:** Results of confirmatory factor analysis.

Model	*χ* ^2^	df	*χ*^2^/df	CFI	TLI	RMSEA	SRMR
Model 1	559	224	2.496	0.942	0.934	0.071	0.041
Model 2	1,201	227	5.291	0.831	0.811	0.121	0.097
Model 3	1,662	229	7.258	0.751	0.725	0.146	0.125
Model 4	3,556	230	15.461	0.422	0.364	0.222	0.228

CFI, comparative fit index; TLI, Tucker–Lewis index; RMSEA, root mean square error of approximation; SRMR, standardized root mean square residual.

Model 1: platform leadership, career centrality, thriving at work, proactive career behavior.

Model 2: platform leadership, career centrality, thriving at work+proactive career behavior.

Model 3: platform leadership + career centrality, thriving at work + proactive career behavior.

Model 4: platform leadership + career centrality + thriving at work + proactive career behavior.

In addition, we examined alternative measurement models to test that our hypothesized model was preferable. First, we examined a three-factor model in which platform leadership, career centrality were influenced by their own factors, whereas thriving at work and proactive career behavior were influenced by another factor (Model 2). However, it was not acceptable (*χ*^2^/df = 5.291; CFI = 0.831; TLI = 0.811; RMSEA = 0.121; SRMR = 0.097). Second, we examined a two-factor model in which platform leadership, career centrality were influenced by another factor, then thriving at work and proactive career behavior were also influenced by another factor (Model 3). This two-factor model was not acceptable (*χ*^2^/df = 7.258; CFI = 0.751; TLI = 0.725; RMSEA = 0.146; SRMR = 0.125). Third, we examined a single-factor model in which all items were loaded on one factor (Model 4). This single-factor model was not acceptable (*χ*^2^/df = 15.461; CFI = 0.422;TLI = 0.364; RMSEA = 0.222; SRMR = 0.228). These findings suggest that measures reported by team members are distinguishable.

### 4.2 Descriptive and correlations

Table 2 presents descriptive statistics among research variables, including mean, standardized deviation and correlations. Platform leadership is positively related with thriving at work (*r* = 0.309, *p* < 0.01), proactive career behavior (*r* = 0.291, *p* < 0.01). Thriving at work are positively related with proactive career behavior (*r* = 0.355, *p* < 0.01). The results align with our theoretical expectations, providing preliminary support for our hypotheses.

**Table 2:** Descriptive statistics among research variables.

Variable	M	SD	1	2	3	4	5	6	7
1 Gender^a^	1.708	0.456							
2 Age^b^	3.157	1.082	0.052						
3 Education^c^	1.816	0.618	0.136^*^	−0.115^*^					
4 Proactive personality	3.505	0.559	−0.192^**^	−0.024	0.094				
5 Platform leadership	4.089	0.613	−0.091	−0.132^*^	−0.015	0.314^**^			
6 Career centrality	3.531	0.747	−0.141^*^	−0.131^*^	0.044	0.515^**^	0.355^**^		
7 Thriving at work	4.063	0.632	0.039	−0.071	0.098	0.248^**^	0.309^**^	0.336^**^	
8 Proactive career behavior	3.791	0.626	−0.060	−0.131^*^	0.157^**^	0.201^**^	0.291^**^	0.283^**^	0.355^**^

*N* = 294; *p < 0.05; **p < 0.01.

^a^Gender was coded as 1 = male; 2 = female.

^b^Age was coded as 1 = below 25; 2 = 26–30; 3 = 31–35; 4 = 36–40; 5 = above 41.

^c^Education was coded as 1 = associate degree or lower; 2 = bachelor degree; 3 = master degree or above.

### 4.3 Examining the hypotheses

To examine the hypotheses above, this paper performed a series of regression analyses. In all regression analyses, age, gender, education and proactive personality are controlled. Table 3 presents the results of these analyses.

**Table 3:** Results of regression analysis.

Variable	Thriving at work				Proactive career behavior			
	M1	M2	M3	M4	M5	M6	M7	M8
Gender	0.085	0.089	0.095	0.104	−0.039	−0.035	−0.065	−0.059
Age	−0.063	−0.029	−0.029	−0.005	−0.109	−0.077	−0.089	−0.069
Education	0.055	0.070	0.058	0.060	0.133^*^	0.148^*^	0.116^*^	0.129^*^
Proactive personality	0.258^**^	0.177^**^	0.114	0.101	0.179^**^	0.101	0.097	0.053
Platform leadership		0.259^**^		−0.176		0.248^**^		0.178^**^
Thriving at work							0.316^**^	0.272^**^
Career centrality			0.284^**^	0.211^**^				
Platform leadership× Career centrality				0.404^*^				
*R* ^2^	0.077	0.136	0.135	0.185	0.074	0.128	0.166	0.192
Δ*R*^2^		0.059^**^	0.058^**^	0.108^**^		0.054^**^	0.092^**^	0.118^**^
*F*	6.044^**^	9.083^**^	8.995^**^	9.285	5.733^**^	8.436^**^	11.441^**^	11.348^**^

*N* = 294; *p < 0.05; **p < 0.01.

Model 6 indicates that platform leadership has a significant positive impact on subordinates’ proactive career behaviors (*b* = 0.248, *p* < 0.01), thus supporting Hypothesis 1. Model 2 shows that platform leadership has a significant positive impact on subordinates’ thriving at work (*b* = 0.259, *p* < 0.01), thus supporting Hypothesis 2.

H3 asserts that thriving at work mediates the relationship between platform leadership and proactive career behavior. Model 8 demonstrates that when platform leadership and thriving at work are both included in the regression equation, the relationship between thriving at work and proactive career behavior is significant (*b* = 0.272, *p* < 0.01), whereas the influence of platform leadership is still significant but lesser in magnitude (*b* = 0.178, *p* < 0.01). It suggests thriving at work partially mediates the relationship between platform leadership and proactive career behavior.

To formally test the mediation effect of thriving at work on the association between platform leadership and proactive career behavior, this paper follows the path analytic approach to estimate the mediation effect (Edwards and Lambert, 2007; Hayes, 2013). This paper relies on PROCESS procedure developed by Hayes (2013, Model 4) to achieve this analysis approach and use a bootstrapping method to estimate the mediation effect. Supporting H4, thriving at work has a significant mediation effect on the relationship between platform leadership and proactive career behavior (mediation effect = 0.072, BootSE = 0.060, 95%CI = 0.027–0.130). Thus, H3 is supported.

**Figure 2:**
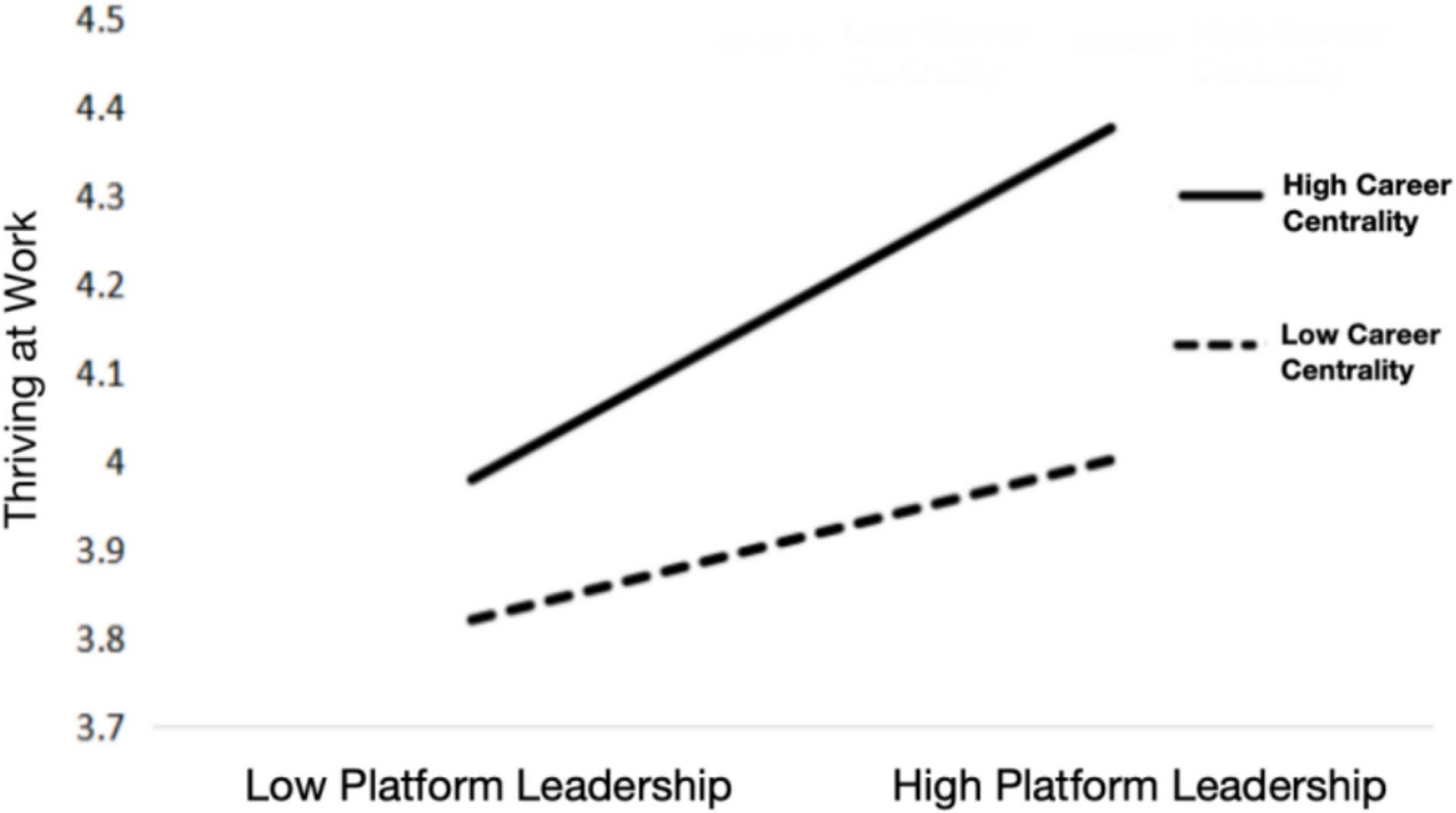
The moderating role of career centrality.

Next, this paper examines the moderating effect of career centrality. We predict thriving at work by including all control variables, platform leadership, career centrality (Model 3) and then additionally include the interaction effect of platform leadership and career centrality (Model 4). Results support that the interaction effect is significant (*b* = 0.404, *p* < 0.05) in predicting thriving at work, supporting H4. Figure 2 presents the interaction plot, which illustrates that the impact of platform leadership on thriving at work is more pronounced among employees with high career centrality. Specifically, when career centrality is high (M + 1 SD), the simple slope of platform leadership on thriving at work is 0.324 (*p* < 0.01), indicating a stronger positive relationship. In contrast, when career centrality is low (M-1 SD), the simple slope is 0.148 (*p* < 0.05), suggesting a weaker but still significant relationship. This finding highlights the importance of career centrality as a moderator in the context of platform leadership and employee thriving.

Finally, this study examines the hypothesized moderated–mediation effects using the PROCESS procedure (Model 7, Hayes, 2013) to estimate the conditional mediation effect of career centrality with a bootstrapping method. The results show that thriving at work had a stronger mediation effect on the relationship between platform leadership and proactive career behavior when career centrality is higher (conditional mediation effect = 0.088, 95%CI = 0.025–0.161) than when career centrality is lower (conditional mediation effect = 0.040; 95%CI = −0.005-0.104). The index of moderated mediation = 0.06 and 95% CI = 0.011–0.093. Thus, H5 is supported.

## 5 Discussion

Our study advances platform leadership and proactive career behavior literature in three ways. First, the results demonstrate that platform leadership enhances subordinates’ thriving at work, which subsequently promotes proactive career behavior. Notably, this thriving-enhancing effect is significantly stronger among employees with high career centrality compared to their low-centrality counterparts. Furthermore, career centrality moderates the indirect pathway through thriving, underscoring its boundary condition role. These findings carry important theoretical and practical implications, as elaborated below.

### 5.1 Theoretical contributions

This study makes three key theoretical contributions. First, it pioneers the integration of platform leadership with career development literature by examining its impact on proactive career behaviors, a previously underexplored outcome. While prior research focused on platform leaderships’ effects on innovation (Hao et al., 2021) and organizational competitiveness (Yang et al., 2022), we extend its scope to career-related outcomes. Our findings validate that platform leadership significantly enhances subordinates’ proactive career behaviors, addressing a critical gap and enriching individual-level mechanisms in this domain. This aligns with broader evidence on leadership as a situational antecedent to career success (Wang et al., 2019, 2024), while offering novel insights into how leaders shape career trajectories through shared platforms.

Second, drawing on Self-Determination Theory (Deci and Ryan, 2000, 2004), we identify thriving at work, a psychological state of vitality and learning (Spreitzer et al., 2005), as the mediating mechanism linking platform leadership to proactive career behaviors. Thriving reflects employees’ cognitive-affective perception of career growth and serves as the precursor to career initiative-taking. Specifically, platform leadership fosters thriving by co-creating business platforms that embed challenge and meaning into work roles. This environment enables employees to recognize the developmental value of their work, thereby motivating proactive career investment. Our mediation findings not only corroborate Hao et al.’s (2021) observations but extend them by delineating the psychological process through which leadership translates into behavioral outcomes.

Third, this study advances an individual-situational interaction perspective by revealing career centrality as a critical boundary condition. We demonstrate that the positive effect of platform leadership on thriving, and consequently on proactive career behaviors, is significantly amplified among employees who prioritize career goals (Erdogan et al., 2018). This suggests that platform leadership’s efficacy depends on followers’ career valuation. For high-centrality employees, the leadership’s developmental signals resonate more powerfully, creating a constructive situational fit. By addressing the “for whom” question, our research answers calls to examine individual moderators in leadership studies (Wang et al., 2019) and provides a nuanced framework for understanding differential follower responses.

Collectively, these contributions deepen theoretical understanding of platform leadership’s mechanisms and contingencies while offering actionable insights for fostering career growth through leadership practices (Table 4).

**Table 4:** Moderated mediation effect.

Mediator	Moderator	Effect	BootSE	95%CI Lower	95%CI Upper
Thriving at work	Career Centrality (-1SD)	0.040	0.028	−0.005	0.104
	Career Centrality (+1SD)	0.088	0.035	0.025	0.161
	Deviation	0.044	0.020	0.011	0.093

### 5.2 Practical implications

This study yields significant practical implications for organizational leadership. First, the significant yet moderate effects of platform leadership on proactive career behaviors (*b* = 0.248) and thriving (*b* = 0.259) suggest targeted implementation yields measurable returns. Platform leadership, through the construction of shared career platforms, can effectively motivate subordinates to proactively manage career trajectories while enhancing their competencies. Leaders demonstrating platform behaviors, such as inclusiveness, empowerment, and resource provision, create environments that satisfy employees’ psychological needs, thereby elevating thriving and catalyzing career initiative-taking. As extrapolated from our coefficients, training managers in platform leadership behaviors (e.g., resource provision) could increase proactive career engagement by 12–15%. Organizations should prioritize platform leadership training for managers overseeing career-central teams to maximize ROI. Such reciprocal growth relationship enhances employees’ likelihood of career success while helping organizations build long-term human capital advantages through talent development and retention (Hao et al., 2021).

Second, this study underscores the pivotal role of career centrality in shaping the effectiveness of platform leadership. The impact of platform leadership on employee thriving is significantly stronger among employees with higher career centrality (*b* = 0.404), indicating that career centrality acts as an important moderator in this relationship. Employees with higher career centrality actively engage with the resources and opportunities provided by their leaders, which in turn enhances their career success. Based on this, organizations should consider career centrality as a key factor in recruitment and selection processes, prioritizing candidates who demonstrate a strong focus on career development. Additionally, targeted career development programs should be implemented to enhance the career awareness and motivation of existing employees, especially those with lower career centrality. This approach can help maximize the benefits of platform leadership and foster a thriving workforce.

Third, at the organizational level, platform leadership can generate broader effects through top-down mechanisms. The implementation of this leadership style relies not only on the initiatives of senior leaders but also on social learning processes that encourage imitation among middle and lower-level managers. This creates an atmosphere of mutual growth throughout the organization. Such a trickle-down effect further amplifies the impact of platform leadership, ensuring that leaders and employees at all levels collaborate on a shared career platform for synergistic development.

### 5.3 Limitations and future directions

First, although this study collected data at multiple time points, it is essentially cross-sectional in nature. As such, our findings can only confirm correlations between variables, not causal relationships. To establish causality more robustly, future studies should employ experimental designs. The overrepresentation of female participants (70.70%) in our sample may limit the generalizability of findings to gender balanced populations, particularly given potential gender differences in career centrality perceptions. Future studies should control for gender distribution or explicitly test gender’s moderating role to clarify its contextual influence. Additionally, all data were collected via self-reports from employees. Future studies should incorporate peer or family assessments to measure employees’ proactive career behaviors.

Second, while we confirmed the effect of platform leadership on subordinates’ proactive career behaviors, as a new leadership type, it is essential to control for other leadership factors, such as transformational leadership and servant leadership, to test the unique predictive validity of platform leadership. Furthermore, since platform leadership emphasizes mutual growth between leaders and subordinates, future longitudinal studies could examine whether exhibiting platform leadership behaviors contributes to leaders’ own career growth and success, thus providing a more comprehensive evaluation of its effectiveness.

Third, this study posits that platform leadership influences proactive career behaviors through the mechanism of thriving at work. However, thriving at work only partially mediates this relationship, indicating that unexamined mediating mechanisms may exist. For instance, perceived career opportunities, which reflect individuals’ perceptions of the availability of work opportunities (Kraimer et al., 2011), warrant further exploration in future research. Additionally, while this study examined the moderating role of career centrality, more individual-level boundary conditions remain to be investigated. For example, learning goal orientation, which reflects individuals’ tendencies to develop competencies by acquiring new skills and mastering new situations (VandeWalle, 1997), might strongly align with platform leadership’s emphasis on learning and growth, thereby positively influencing career behaviors. Lastly, our findings may be contextualized by China’s high power distance and collectivist norms (Hofstede et al., 2010). Future research should explicitly test how these cultural dimensions moderate the observed relationships. For instance, whether power distance amplifies leaders’ platform building legitimacy or collectivism reinforces career centrality’s role in goal internalization.

## 6 Conclusion

Platform leadership has positive effects on numerous outcomes at the individual, team, and organizational levels. However, this study is the first to investigate the relationship between platform leadership and subordinates’ career behaviors. This study examines the relationship between platform leadership and subordinates’ proactive career behaviors, as well as its underlying mechanisms. The results demonstrate that platform leadership positively influences proactive career behaviors through thriving at work. Furthermore, career centrality enhances the positive effect of platform leadership on thriving at work and amplifies the mediating role of thriving at work in the relationship between platform leadership and proactive career behaviors. This study contributes to the literature on platform leadership and career behaviors, providing valuable insights for leadership training and development practices.

## Data Availability

The raw data supporting the conclusions of this article will be made available by the authors, without undue reservation.
